# Frailty and health related quality of life in older Mexican Americans

**DOI:** 10.1186/1477-7525-7-70

**Published:** 2009-07-23

**Authors:** Meredith C Masel, James E Graham, Timothy A Reistetter, Kyriakos S Markides, Kenneth J Ottenbacher

**Affiliations:** 1 The University of Texas Medical Branch Galveston 301 University Blvd Route 1137, Galveston, TX 77555-1137, USA

## Abstract

**Background:**

Previous research on frailty in older adults has focused on morbidity and mortality. The purpose of this study was to elicit the relationship between being non-frail, pre-frail, or frail and health related quality of life in a representative sample of older Mexican Americans surveyed in 2005–2006.

**Methods:**

Data were from a representative subsample of the Hispanic Established Populations Epidemiologic Studies of the Elderly (EPESE) and included 1008 older adults living in the community (mean (sd) age = 82.3(4.3)). Multiple regression analyses examined the relationship between frailty status and the eight SF-36 health related quality of life subscales and two summary scales. Models also adjusted for the participants' sociodemographic and health status.

**Results:**

We found that, after adjusting for sociodemographic and health related covariables, being pre-frail or frail was significantly associated (p < 0.001) with lower scores on all physical and cognitive health related quality of life scales than being non-frail.

**Conclusion:**

When compared to persons who are not frail, older Mexican American individuals identified as frail and pre-frail exhibit significantly lower health related quality of life scores. Future research should assess potential mediating factors in an effort to improve quality of life for frail elders in this population.

## Background

Frailty is a state of pre-clinical disability, making a person more susceptible to functional decline [1] and adverse health outcomes including disability, falls, and institutionalization [2-4]. In addition, the health of frail older adults limits the amount and scope of activities that they perform [5]. These poor outcomes, in turn, can have negative implications on health related quality of life (HRQOL) [6-8]. Health related quality of life, however, involves more than a self-assessment of functional status; it also conveys an individual's sense of satisfaction with that level of functioning [9] relative to his or her unique circumstances and values.

Both frailty and HRQOL vary between individuals with similar health conditions as well as within the same individual over time [10,11]. Both concepts are also widely used without consensus definitions but are generally acknowledged to result from the interaction of multiple systems and/or domains [10,12]. Despite these similarities, HRQOL has not been examined in a population of older adults using an operationalized index of frailty except in smaller qualitative studies [5]. Puts and colleagues recently reported that among a smaller group of community-dwelling older adults, those who were frail reported worse health-related quality of life than those who were non-frail. The authors suggested that a larger study could confirm the findings [5].

The current study examines the relationship between frailty and self-reported HRQOL in older Mexican Americans while adjusting for select sociodemographic characteristics and health factors. Older Mexican Americans comprise one of the fastest growing segments of the U.S. population [13], yet no study could be found pertaining to the impact of frailty on HRQOL in this group. We hypothesized that frailty status would be associated with decreased HRQOL and that the physical aspects of HRQOL would demonstrate stronger relationships with the frailty index scores than the mental aspects of the HRQOL measure.

## Methods

### Study Population

Data were from a sub-sample of the Hispanic-Established Populations for the Epidemiological Study of the Elderly (EPESE) who participated in an investigation related to the development of frailty. The Hispanic EPESE is a longitudinal study of Mexican Americans residing in Texas, New Mexico, Colorado, Arizona and California. Study participants were originally identified by area probability sampling procedures that involved selecting counties, census tracts, and households within defined census tracts. The sampling procedure assured a sample generalizable to approximately 500,000 older Mexican Americans living in the southwest in the early 1990s and has been previously described in detail [14].

The current study included 1,013 community-dwelling Mexican Americans, ages 74 years and older, who participated in the Frailty Study in 2005–2006. The inclusion criteria were the ability to perform the items necessary to complete an operationally defined measure of frailty [1] and a standardized assessment of HRQOL [15] (description below). No data obtained through proxy were permitted. The final sample size for the analyses was 1008. Participants were interviewed and examined in their homes by raters who received 20 hours of training in assessments of physical functioning including balance, gait, and functional daily living skills and HRQOL. Interviews were conducted in Spanish or English, depending on the participant's preference. Fifteen percent of each interviewer's work was validated by follow-up telephone contact. The University's Institutional Review Board on human protection and research ethics approved the study.

### Study Variables

*Health Related Quality of Life (HRQOL) *was measured using the Medical Outcomes Study (MOS) Short Form – 36 (SF-36) [15]. The SF-36 is comprised of eight subscales measuring physical functioning, daily activity limitations, bodily pain, general health, vitality, social functioning, and mental health. Scores from each subscale are standardized and range from 0–100, with higher scores indicating positive self-assessment. In addition, there are two composite scales that summarize the physical and mental components of the SF-36. The Physical Component Scale (PCS) ranges from 0–100 with 100 indicating absence of physical problems, high energy, and excellent self-rated health [16]. The Mental Component Scale (MCS) also ranges from 0–100 with 100 indicating no difficulties or impairments in daily functioning due to psychological issues [16]. The use of the SF-36 in measuring HRQOL in older Mexican Americans has been previously validated [17].

*Frailty *was measured using a modified version of the index developed by Fried and colleagues [1]. Hand grip strength, exhaustion, physical activity, unintended weight loss, and walking speed were used to create a five-point index of frailty symptoms. One point was assigned if a participant 1) scored in the bottom quartile for hand grip strength (adjusted for gender and BMI), 2) had greater than or equal to 10 pounds of unintended weight loss in the previous year, 3) scored in the bottom quintile for walking speed (adjusted for gender and height), 4) reported a moderate or greater amount of time feeling exhausted during the prior week (as determined by responses to the Centers for Epidemiologic Study-Depression scale (CES-D)) [18], or 5) scored in the bottom quintile for exercise (adjusted for gender) as measured by the *Physical Activity Scale for the Elderly *[19]. The *Physical Activity Scale for the Elderly *has been previously validated and deemed appropriate for use in studies of community-dwelling adults, even those who are sedentary [19,20]. Participants with a zero score were considered non-frail, those with one to two symptoms were considered pre-frail, and those with three or more symptoms were considered to be frail.

There are two areas of difference between our Frailty Index and the original index created by Fried and colleagues[1]. First, to assess activity level Fried and colleagues used the *Minnesota Leisure Activity Questionnaire *[21] and we used the *Physical Activity Scale for the Elderly *[19]. Second, we did not use the actual cut point scores developed by Fried and others since the sample in their study was younger than our baseline sample, and anthropometric values (weight and height), used to adjust for handgrip muscle strength and walking speed, are known to differ in Mexican Americans compared to the predominantly non-Hispanic white sample included in the Fried and colleagues' original frailty study[1]. When analyzing frailty in samples different from those in the Cardiovascular Health Study, Fried and others [22,23], have also used slightly different criteria or cut-points to construct the frailty index.

*Sociodemographic and health-related covariables *included participants' age, sex (male = 0, female = 1), marital status (married = 1, not married = 0), as well as select measures of socioeconomic status. Education level was measured by number of years of schooling ranging 0–20 years. Financial strain was measured by asking participants how much difficulty they had paying monthly bills (no trouble, a little, some, or a great deal of difficulty (range 0–3)).

Participants' health was also measured by their response (no = 0, yes = 1) to self-reported doctor diagnosis of arthritis, heart attack, stroke, hypertension, cancer, diabetes, hip fracture, or other fractures. All comorbidities with the exception of arthritis were combined to create a comorbidity index, whereas arthritis was included in the analyses independently because of its particularly strong relationship to frailty and certain subscales of the SF-36 in preliminary analyses. In addition, body mass index was calculated by dividing individuals' weight in kilograms by height in meters squared. Body mass index (BMI) categories (underweight, normal weight, overweight, or obese) as defined by National Center for Chronic Disease Prevention and Health Promotion at the United States' Centers for Disease Control were used in the analyses.

### Data Analysis

*Statistical analyses *were carried out using SAS 9.1 software [24]. Baseline descriptive statistics were presented by frailty category and differences between groups were assessed via ANOVA and chi-square tests for independence. Differences in mean scores on the SF-36 subscales by frailty category were also identified using ANOVA. Multivariable models testing the effect of frailty category on the SF-36 subscale scores were conducted using multiple linear regressions. In addition, logistic regression was used to estimate odds ratios for the effect of frailty status on being in the lowest quartile of the SF-36 summary scales (PCS and MCS). Regression diagnostics included tests for linearity between the predictor and outcome variables and tests for normality of residuals with kernel density plots and found no violations of basic assumptions for regression. Model fit statistics were examined to assure goodness of fit (results not presented).

## Results

Table 1 presents baseline characteristics of the participants stratified by frailty status. The sample consisted of 264 (26%) non-frail participants, 547 (54%) participants who were pre frail, and 200 (20%) participants characterized as frail. Most participants were in their early 80s, and were female. Over one-third of participants experienced some or a great deal of difficulty paying bills and most had less than a 6th grade education. Less than half were married. With regard to health conditions, a majority reported being diagnosed with arthritis and had one or two other chronic medical conditions. In addition, over half of the sample was overweight or obese as measured by Body Mass Index.

**Table 1:** Baseline characteristics of participants in the Frailty subsample of the Hispanic EPESE (n = 1008) by frailty status.

	**Not Frail**	**Prefrail**	**Frail**
	(n = 264)	(n = 547)	(n = 200)
	M (SD)/%	M (SD)/%	M (SD)/%
**Sociodemographic Covariables**			
Age*	81.2 (4.0)	82.5 (4.6)	83.0 (5.0)
Female	61.4	62.7	67
Education*	5.1 (4.0)	5.3 (4.0)	4.5 (3.3)
Married*	46	41	34
Financial Strain (some or a great deal)*	37.8	42.5	30.5
**Health-Related Covariables**			
Arthritis*	52	64	77
Chronic Illnesses*	1.5 (1.1)	1.8 (1.2)	2.3 (1.3)
BMI	26.8 (4.4)	27.6 (5.1)	27.4 (5.4)
Underweight*	1.3	0.4	4
Normal Weight	36.3	31.8	32
Overweight*	41.2	41.6	34
Obese	21.2	26.2	30
**Health-Related Quality of Life (SF-36)**			
General Health Perception*	68.3 (17.3)	57.7 (20.4)	43.5 (20.9)
Physical Function*	64.3 (27.7)	44.5 (30.5)	23.3 (24.2)
Role: Physical*	80.6 (37.0)	54.0 (47.1)	31.4 (43.2)
Pain*	77.2 (24.8)	64.1 (29.5)	49.7 (31.0)
General Mental Health*	84.8 (14.9)	78.0 (19.6)	66.2 (21.4)
Role: Emotional*	94.6 (20.9)	78.1 (40.2)	52.8 (48.3)
Vitality*	72.9 (18.4)	60.6 (22.1)	44.6 (22.6)
Social Function*	88.6 (20.7)	71.6 (30.5)	47.8 (33.8)
Physical Component Scale*	44.1 (10.4)	36.2 (11.9)	29.1 (9.9)
Mental Component Scale*	58.4 (6.3)	54.5 (10.7)	46.9 (12.7)

*significantly different means or proportions by frailty status (p < 0.05) as determined by ANOVA and chi-square tests of independence

In most cases, with the exception of gender and BMI, the characteristics of the sample differed by frailty category. For example, those who were frail were older and had greater prevalence of arthritis and chronic illnesses. The same pattern emerged for quality of life scores on the SF-36 scales in that those who were frail had lower scores than the non-frail participants (see Table 1).

Table 2 provides standardized regression coefficients for the effect of frailty category on the subscales and summary scales of the SF-36 quality of life measure. On all subscales and both the physical and mental summary scales, being pre-frail or frail was associated with lower scores.

**Table 2:** Multiple regression coefficients (standardized) for the effect of frailty category on SF-36 Scales in the frailty subsample of the Hispanic EPESE (n = 1008).

Frailty Category	General Health Perception	Physical Function	Role: Physical	Pain	General Mental Health	Role: Emotional	Vitality	Social Function	Physical Component Scale	Mental Component Scale
Prefrail	**-0.20**	**-0.24**	**-0.25**	**-0.18**	**-0.16**	**-0.20**	**-0.22**	**-0.24**	**-0.26**	**-0.18**
Frail	**-0.41**	**-0.44**	**-0.38**	**-0.26**	**-0.33**	**-0.38**	**-0.45**	**-0.49**	**-0.42**	**-0.40**
Age	0.03	**-0.14**	-0.06	0.03	0.07	0.04	**-0.01**	-0.05	**-0.10**	**0.09**
Female	**-0.09**	**-0.18**	**-0.09**	**-0.10**	**-0.12**	-0.04	-0.10	-0.06	**-0.15**	-0.04
Education	**0.09**	**0.09**	**0.10**	**0.09**	0.04	0.07	0.06	**0.08**	**0.11**	0.04
Married	-0.04	0.03	-0.01	0.003	0.01	-0.003	-0.01	-0.04	0.005	-0.02
Low Financial Strain	0.04	**0.09**	0.01	0.06	0.07	0.07	0.05	**0.08**	0.05	0.07
Arthritis	**-0.14**	**-0.14**	**-0.10**	**-0.25**	**-0.12**	-0.07	**-0.13**	**-0.10**	**-0.19**	**-0.07**
Chronic Illnesses	**-0.11**	**-0.10**	**-0.08**	**-0.12**	-0.07	-0.04	**-0.09**	**-0.13**	**-0.12**	-0.07
Underweight	0.002	0.005	0.03	-0.02	-0.01	0.05	0.04	-0.03	0.004	0.02
Overweight	0.05	-0.02	0.005	0.002	-0.01	0.03	0.01	0.06	0.003	0.03
Obese	0.01	-0.07	-0.06	-0.02	-0.04	0.02	-0.03	0.04	-0.06	0.02

Notes: **Bold **values are significant at p < 0.05; The reference category for frailty status was "Not Frail", and the reference category for Body Mass Index was "Normal Weight"

Logistic regressions were employed to establish the odds of scoring in the lowest quartile on the SF-36 summary scales. Table 3 displays the results of the logistic regression analyses. Even in the presence of sociodemographic and health-related covariables, being pre-frail was associated with approximately four times the odds of having a physical or mental component score in the bottom quartile of the sample than those who were not frail. Furthermore, frail participants had approximately 10 times the odds of scoring in the bottom quartile of either scale than their non-frail counterparts.

**Table 3:** Odds ratios for the effect of frailty status on scoring in the lowest quartile of the SF-36 summary scales in the frailty subsample of the Hispanic EPESE (n = 1008)^a^

	PCS		MCS	
	OR	(95% CI)	OR	(95% CI)
Not Frail	1.00		1.00	
Prefrail	4.03	(1.95, 8.35)	3.86	(2.07, 7.19)
Frail	10.58	(4.90, 22.84)	10.20	(5.19, 20.07)

^a ^Adjusted for age, sex, marital status, financial strain, arthritis, chronic illnesses, and BMI

Several sensitivity analyses were conducted to eliminate potential study limitations. *Activities of Daily Living *(ADL) and the *Centers for Epidemiologic Study-Depression *scale (CES-D)[18], for example, are measures included in the Hispanic EPESE, and both are associated with frailty. However, in an effort to avoid redundancy with the outcome measures, they were excluded from the current analyses. ADL measures and those evaluating depressive symptoms in the CES-D are too closely related to questions from the physical function and mental health subscales of the SF-36 to include them in a well-fitted statistical model. Nevertheless, we tested the models with ADL and CES-D measures and this did not alter our findings (data not shown).

Because of the strong relationships between gender, arthritis, and both frailty and the SF-36, interactions between gender and frailty status as well as arthritis and frailty status were also examined. No significant interactions were found (data not shown).

## Discussion

Physical frailty in older adults is a risk factor for numerous detrimental outcomes such as mortality, cardiovascular disease, and disability[1,25]. Until now, research on frailty has largely ignored the effect of frailty on psychosocial outcomes such as health related quality of life. In order to explore the extent to which frailty permeates a person's life, we examined the relationship between a commonly used index of frailty and quality of life indicators in a sample of older Mexican Americans.

We found that being pre-frail or frail was significantly associated with lower scores on perceptions of general health, physical function, bodily pain, physical and emotional roles, mental health, vitality, and social function on the SF-36 HRQOL measure compared to those who were non-frail (see Tables 1 and 2). Furthermore, both pre-frail and frail states were associated with greater odds of scoring in the lower quartile of the mental and physical component scales of the SF-36 relative to participants categorized as non-frail (see Table 3).

The finding that a standardized measure of frailty can differentiate quality of life ratings in aging Mexican Americans is important for two reasons. First, low scores on the mental and physical component summary scales are indicators of considerable physical limitations and repeated psychological distress[16]. In addition, lower scores on items such as the general health subscale have been associated with greater hospitalizations, more doctor's office visits, and greater numbers of prescriptions [26]. Second, previous research has shown that frailty is a dynamic state that is responsive to focused interventions [3,25]. Thus, better detection, management, and prevention of frailty in older adults may have desirable effects on both perceived HRQOL and health care utilization among aging older adults.

Previous researchers [27] have suggested that a limitation of the frailty index proposed by Fried and colleagues is that it lacks cognitive measures thus making it incomplete. With these data, however, we have shown through a relationship between frailty and the SF-36 cognitive items that although there are no specific measures of cognitive health in the frailty index, the measures imply a cognitive component. In other words, it may be unnecessary to add explicit cognitive items to the frailty scale to elicit a relationship between frailty and mental/psychosocial status as assessed by the SF-36.

Our findings suggest and support the need for continued research on interventions that address psychosocial, physical and cognitive approaches to improved health related quality of life. Cognitive approaches with older adults have been shown to lessen the likelihood of declines in HRQOL [28], and may also be useful and beneficial within the older Hispanic population to protect against declines in HRQOL in those who are pre-frail or frail. In the ACTIVE clinical trial of cognitive memory and reasoning among older adults, researchers not only found improvement in cognitive abilities but also gains in mobility and health related quality of life [29]. Similarly, researchers have stated that physical activity programs improve function among older adults [30]. It is plausible to suggest that similar quality of life changes could be obtained among older Hispanics who participate in physical programs. Finally, in frail adults, where physical interventions are not practical, psychosocial factors and family support may be the proper intervention to positively influence health among older Hispanics. In a study of Mexican Americans with diabetes, Wen and colleagues found that the presence of family support was associated with better health behavior [31]. There is need to further examine the use of physical, cognitive, and social interventions to improve HRQOL and protect older Hispanics from becoming frail.

Our study includes several strengths. To our knowledge, this study is the first to examine HRQOL in relation to frailty. We collected data from a large population-based sample of Mexican American older adults who represent the fastest growing segment of the aging population. Furthermore, the data were collected prospectively by investigators with experience in community-based research using a well established and validated measure of HRQOL, the SF-36. Examining the effects of frailty on psychosocial outcomes rather than physical outcomes or mortality is unique and contributes to a broader understanding of frailty [32].

Study limitations included the ethnic homogeneity of our sample as well as the cross-sectional approach of our analyses, which decrease the generalizability of the current findings. Another limitation is the self-report nature of several key variables. Furthermore, although the *Physical Activity Scale for the Elderly *is an appropriate measure to assess activity in community-dwelling older persons, it has not explicitly been validated in the Mexican American population. It is possible, therefore, that the scale does not elicit the correct information because of cultural differences from the populations in which it was validated.

## Conclusion

In sum, being pre-frail or frail was strongly associated with diminished health related quality of life in a large sample of Mexican American older adults. Future research on health related quality of life in this population should consider physical frailty as a contributing factor. In addition, gender or disease-specific studies that more closely examine health related quality of life within frailty groups (pre-frail or frail) might help to explain the basic relationship. Furthermore, interventions to prevent, delay, or reverse the cycle of frailty may also have a beneficial impact on the health related quality of life of participants.

## Competing interests

Drs KO and KM were principal investigators or co-investigators on the grants that funded this research (see sources of funding below).

## Authors' contributions

MM conducted the statistical analyses and drafted the manuscript. JG contributed to the literature review, analyses, and final approval of the submitted manuscript. TR contributed to crafting the discussion section and final approval of the manuscript. KM was involved in data collection, data analyses, and final approval of the manuscript. KO was responsible for the data, contributed to the analysis plan, and read and approved the final manuscript.
